# Effect of Laser Beam Oscillation on Laser Welding–Brazing of Ti/Al Dissimilar Metals

**DOI:** 10.3390/ma12244165

**Published:** 2019-12-11

**Authors:** Xi Chen, Zhenglong Lei, Yanbin Chen, Yu Han, Meng Jiang, Ze Tian, Jiang Bi, Sanbao Lin, Nan Jiang

**Affiliations:** State Key Laboratory of Advanced Welding and Joining, Harbin Institute of Technology, Harbin 150001, China; chenxi_hit2016@163.com (X.C.); 18245037440@163.com (Y.H.); mengjiang@hit.edu.cn (M.J.); tztzcool@163.com (Z.T.); bijiang198905@163.com (J.B.); jiangnanhit@yeah.net (N.J.)

**Keywords:** dissimilar metals, Ti/Al butt joint, laser beam oscillation, microstructure, tensile properties

## Abstract

Ti4Al6V and 6061 Al dissimilar metals were butt welded by the laser oscillating welding method. The effects of laser offset, oscillation frequency, and energy distribution on the formation, microstructure, and tensile properties of dissimilar metal joints are discussed in detail. The experimental results show that the Ti6Al4V was micro melted with a laser offset of 1.1 mm, and a large number of intermetallic compounds (IMCs) were formed on the side of the Ti6Al4V. Additionally, there were some porosity defects in the fusion zone (FZ) due to an inappropriate laser oscillation frequency. When the laser offset was increased to 1.2 mm, the IMC distribution was uniform and the thickness was controlled below 2 μm. The porosity defects in the FZ decreased and the tensile strength of the joints increased significantly. The maximum value of tensile strength reached 173 MPa at a laser frequency of 28 Hz.

## 1. Introduction

Low-density hybrid components consisting of dissimilar metals such as Ti/Al, Al/steel, Ti/Mg, and Mg/steel have excellent material properties and consequently find applications in automobile, aerospace, rail transportation, and other fields [1,2,3,4]. Combining the comprehensive advantages of both metals results in hybrid materials fulfilling the specific application requirements of industry [5,6,7]. However, achieving a reliable connection between two dissimilar metals is a difficult task due to their significant differences in physical, chemical, and metallurgical properties [8,9,10]. Therefore, it is becoming a hot research topic to find a reliable and efficient process for joining dissimilar metals.

Many researchers have investigated joining technology among dissimilar metals. Various methods, including laser welding [11,12,13], laser melting deposition [14], laser-arc hybrid welding [15], tungsten inert gas (TIG) [16], metal inert gas (MIG) [17], friction stir welding [18], and ultrasonic spot welding [19] have been studied. Currently, to weld two dissimilar metals, a welding–brazing process is followed, in which a welded joint is formed on the side of the metal with a lower melting point, whereas a brazed joint is formed on the side of the metal with a higher melting point. In order to obtain high-performance welded joints, it is particularly important to control the growth of intermetallic compounds (IMCs) at the brazing interface. Due to high energy, high cooling rate, and fast welding, the laser welding–brazing technique is recommended for joining dissimilar metals. Chen et al. [20] studied the welding of 5052 Al and Q235 steel via laser-CMT (cold metal transfer) process and investigated the effect of process parameters on the mechanical properties and microstructure of the hybrid material. It was observed that the tensile strength decreased with increasing wire feed speed, with the maximum value reaching 83.4 MPa. Tomashchuk et al. [21] successfully joined 5754 Al and T40 Ti with a double-beam laser welding process and studied the influence of opening angles on the IMC formation and the tensile properties of welding joints. Chen et al. [22] used a combination of simulation and experimental methods to study the interfacial reactions of the welding–brazing process of Ti/Al dissimilar metals and found that the V-shaped groove and rectangular spot laser were more conducive to regulating the uniform growth of IMCs. 

In order to increase the mechanical properties of joints, researchers have started applying novel techniques, in addition to the adjustment of the process parameters of the laser welding–brazing process, to regulate the growth of IMCs. Tan et al. [23,24] added a Ni coating onto a Ti surface to improve the mechanical properties of Mg/Ti joints and also suggested a mechanism for improvement. Sun et al. [25] studied the effect of a magnetic field on the formation of IMCs in Ti/Al dissimilar joints. Guo et al. [26] investigated the effect of a TC4 remelting process on the tensile strength of welded joints, and found that, due to the solute redistribution process, the tensile strength increased from 142 to 165 MPa. Wang et al. [27] studied the effect of Zn foil addition on the formation of IMCs and the mechanical properties of joints for the welding of Ti6Al4V and Al5052 via TIG welding. It can be inferred from the earlier studies that the growth of IMCs can be significantly regulated by optimizing the composition and heat source distribution. Consequently, the mechanical properties of joints can be further improved.

In this manuscript, Ti6Al4V and AA6061 sheet metals were joined using a welding method based on laser oscillation using optimized energy distribution. The effects of laser offset and oscillation frequency on the formation, microstructure, and tensile properties of the dissimilar Ti/Al joints were investigated in detail. The aim of this manuscript was to obtain the welding characteristics of the laser oscillating welding process for Ti/Al dissimilar metals.

## 2. Experimental Procedures

**Figure 1:**
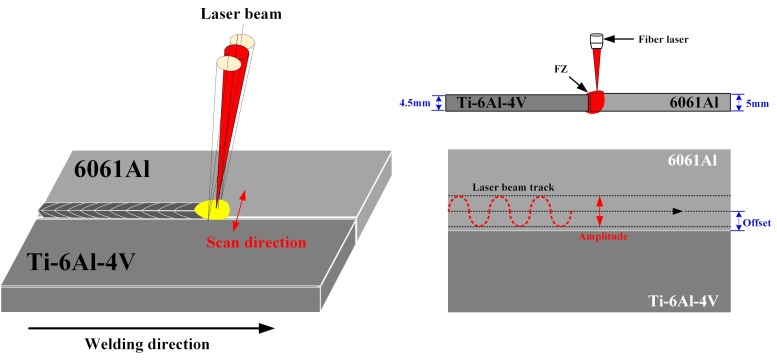
Schematic diagram of laser welding of Ti/Al via beam oscillation.

AA6061 and Ti6Al4V sheets with thicknesses of 5 mm and 4.5 mm, respectively, were obtained from Shanghai Meijun Metal Products Co., Ltd (Shanghai, China). Before the welding process, the sheet metals were cleaned by acetone and mechanical grinding for the removal of oil fouling and oxide film from the metal surfaces. The welding–brazing equipment mainly consisted of a 10-kW fiber laser (YLS-10000, IPG, Oxford, MA, USA), a six-axis KUKA robot (KR 60-4 KS-F, Bavaria, Germany), and a ScanTracker laser processing head (YW-52, Precitec GmbH & Co.KG, Baden-Baden, Germany). The wavelength of the laser was 1064 nm and the laser beam diameter was 0.46 mm at the focusing location with the energy having a Gaussian distribution. The Ti/Al dissimilar metals were butt welded according to the process parameters listed in Table 1. The distribution rule of laser power means that, to obtain high-quality Ti/Al welding–brazing joints, the higher laser power should be on the Al alloy to melt the metal and the lower laser power should be on the Ti alloy to preheat the TC4 surface. According to this rule, the focus position of the laser was on the surface of the Al alloy and the laser offset was set at 1.1 mm and 1.2 mm to the TC4 side. The amplitude of the oscillating laser beam (A) was 2 mm and the frequency (f) was set at 25–30 Hz. The welding speed along the length of the bead was 1 m/min. A schematic diagram of the laser welding of Ti/Al via beam oscillation is shown in Figure 1. Ar with a purity of 99.9% was used as the front and back shielding, with the gas flow rate being 15 L/min (front) and 5 L/min (back).

After welding, the samples for micro-structural observation and tensile strength were prepared by a wire-cutting machine. The welding formation and cross-section of Ti/Al dissimilar joints was performed with an optical microscope (OM, VHX-1000, KEYENCE, Tokyo, Japan). The thickness and distribution of the IMC layer was observed with a scanning electron microscope (SEM, Zeiss Merlin Compact, Baden-Württemberg, Germany).

The tensile properties of the welded joints fabricated according to different process parameters were tested on a tensile testing machine (AGX-plus, Instron, Norfolk County, MA, USA) with a strain rate of 2 mm/min at room temperature. Three tensile specimens were selected for each parameter, and the values were averaged. The tensile fractures of different joints were analyzed by SEM and the elemental distribution of the fracture surface was tested by energy dispersive spectroscopy.

## 3. Results and Discussion

### 3.1. Laser Power Input Mode

**Figure 2:**
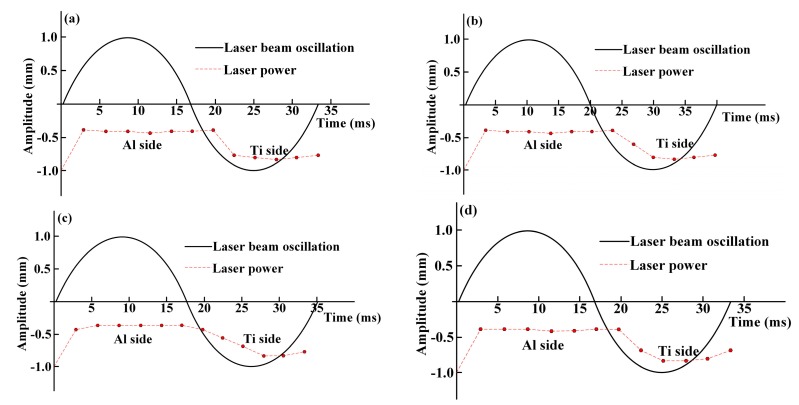
Schematic diagram of laser beam oscillation and laser power distributions: (**a**) 1#; (**b**) 2#; (**c**) 3#; (**d**) 4#.

The effect of input of laser power on the welding process of Ti/Al butt joints via a laser welding process is given in Figure 2. In order to form a stable brazing joint on the side of the Ti6Al4V alloy without melting the Ti6Al4V, a fiber laser was chosen with beam oscillation to join the Ti/Al butt joints. The oscillation amplitude was set at 2 mm and three different frequencies of 25, 28, and 30 Hz were chosen. The laser energy distribution was also controlled so as to have less on the side of the Ti4Al6V alloy, which resulted in preheating of the surface of the Ti4Al6V alloy without melting. The offset of the laser beam to the side of the 6061 Al was set at 1.1 mm in Figure 2a,b, and changed to 1.2 mm in Figure 2c,d. The laser power distributions corresponding to different process conditions are listed in Table 1. In one oscillation cycle, the welding length along the length of the bead is given by L = v_x_/f, where L is the welding length, v_x_ is the welding speed, and f is the oscillation frequency. The spatial overlap of the oscillations is given by φ = d/2L = df/2v_x_. For oscillation frequencies of 25, 28, and 30 Hz, different values of φ are 26.4%, 38.6%, and 41.4%, respectively. The linear energy density along the length of the bead is η = P_a_/v_x_, where P_a_ is the average laser power in one oscillation cycle, P_a_ = (P_1_ + P_2_ + … + P_12_)/12. P_1_, P_2_…P_12_ are the laser powers at the corresponding positions. For the 1#, 2#, 3#, and 4# samples, the linear energy densities were 1.975, 1.958, 2.525, and 2.308 J/mm, respectively.

### 3.2. Weld Formation

**Figure 3:**
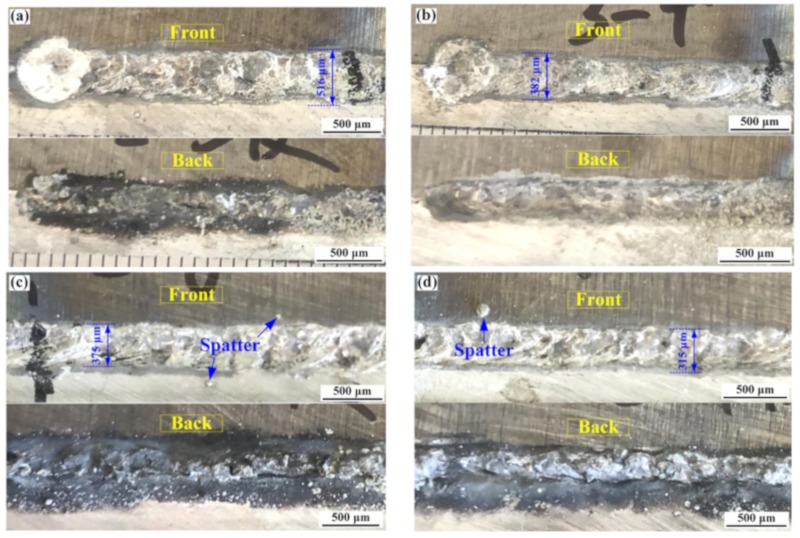
Weld formations of Ti/Al joints produced by different laser offsets and energy distributions: (**a**) 1#; (**b**) 2#; (**c**) 3#; (**d**) 4#.

The weld formations of the Ti/Al butt joints produced by different offsets and laser energy distributions are shown in Figure 3. In Figure 3a,b, it can be seen that the two metals formed tight welding–brazing joints with the laser offset of 1.1 mm. However, with a 1.2 mm laser offset, the welding process was found to be unstable, with some spatters at the edge of the weld. With a 1.1 mm laser offset, the weld formation became more continuous, with the front and back formation of welds improving when the frequency was decreased from 30 to 25 Hz. The weld surface showed metallic luster and a fish-scale shape. The width of the welded joints decreased to 375 and 315 μm when the laser offset was changed from 1.1 to 1.2 mm, respectively. The laser beam oscillation caused intense melting flow which further affected weld formation.

**Figure 4:**
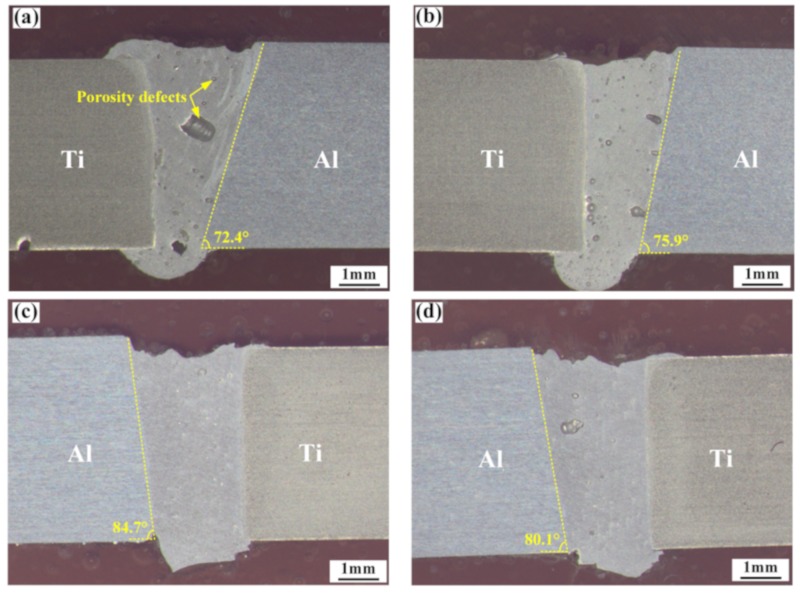
Cross sections of Ti/Al joints produced by different laser offsets and energy distributions: (**a**) 1#; (**b**) 2#; (**c**) 3#; (**d**) 4#.

The cross sections of the Ti/Al welding–brazing joints produced by different laser offsets and energy distributions are given in Figure 4. The observed cross sections indicate that the laser offset has a significant effect on the formation of welded joints. When the laser offset was 1.1 mm, most of the laser energy was absorbed by the aluminum alloy, leaving a small part to be absorbed by the TC4 alloy, which resulted in micro melting of the TC4 alloy. Along the vertical direction of the Al surface, the molten amount of Ti and Al alloys gradually decreased. This is because the gradual absorption of laser energy by the molten pool decays along the vertical direction, resulting in a V-shaped weld (Figure 4a,b). The melting angles on the side of the 6061 Al were 72.4° and 75.9°, respectively. The molten titanium alloy reacted with the aluminum in the weld and a large number of IMCs were formed near the TC4 side. Additionally, some porosity defects were formed in the weld during the solidification process. The porosity defects can be divided into two types: process porosity (induced by keyhole collapse) and metallurgical porosity (volatilization from low-melting-point elements). With a decrease in frequency from 30 to 25 Hz, the size of porosity defects significantly decreased. When the laser offset increased to 1.2 mm, the weld shape changed from V-shaped to U-shaped due to non-melting TC4 alloys, since the laser energy was mostly used for the melting of aluminum. A better welding–brazing joint was formed with a 1.2 mm laser offset. The laser beam oscillation had a stirring effect on the molten pool, which can promote the escape of bubbles from the molten pool [28]. However, with the increase of oscillation frequency, the stability of the keyhole gradually decreased, and the probability of process porosity increased. Therefore, the porosity defects first decreased and then increased with the increase of oscillation frequency. At an oscillation frequency of 28 Hz, the porosity defects almost disappeared.

### 3.3. IMC Distribution

**Figure 5:**
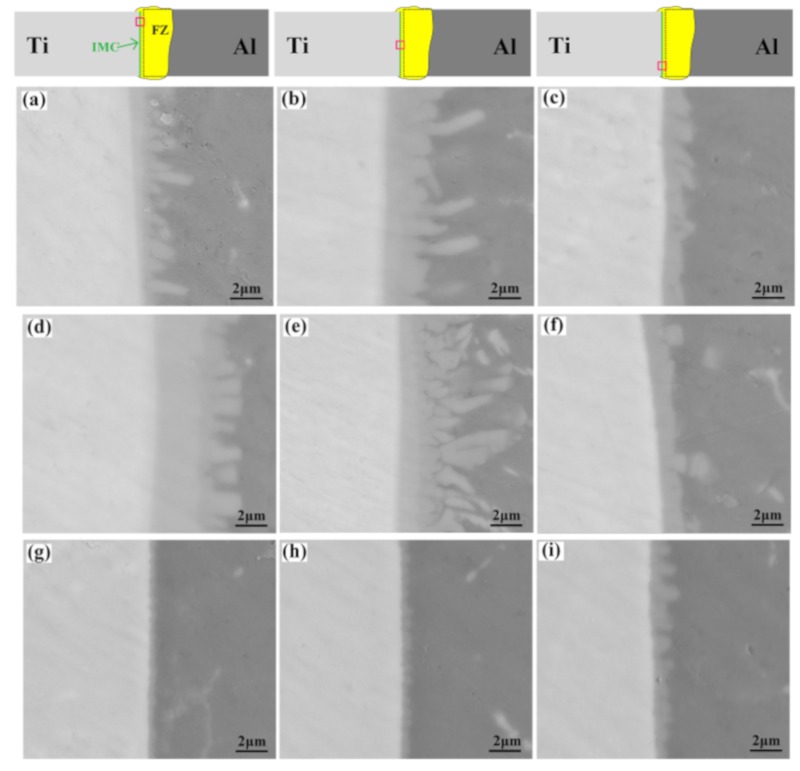

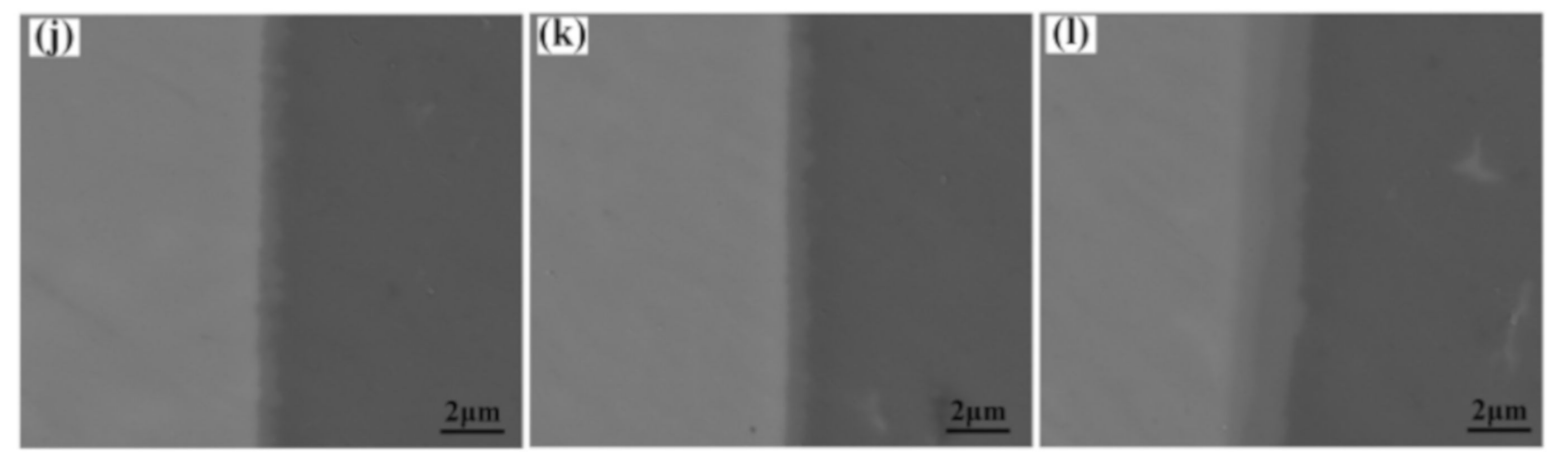
IMCs distribution in Ti/Al joints produced by different laser offsets and energy distributions: (**a**–**c**) 1#, (**d**–**f**) 2#, (**g**–**i**) 3#, (**j**–**l**) 4#. FZ: fusion zone.

As depicted in Figure 4, IMCs formed during the welding process. The TC4 alloy near the weld seam melted slightly when the laser offset was 1.1 mm. In order to better reveal the influence of energy distribution on the growth of IMCs, the IMCs formed in the presence of different process parameters are shown in Figure 5. For the laser offset of 1.1 mm, serrated IMCs were formed along the TC4 surface. The thickness of the IMCs at different positions of the weld was observed to be different. For a 30 Hz oscillation frequency, the thicknesses of the IMCs in the upper, middle, and lower positions of the weld were 3.1, 4.9, and 1.7 μm, respectively (Figure 5a–c). These changed to 5.6, 7.4, and 1.6 μm, respectively, for an oscillation frequency of 25 Hz (Figure 5d–e). When the laser offset was increased to 1.2 mm, the thickness of the IMCs was significantly decreased due to the optimization of laser energy distribution. The thickness of the IMCs at different positions tended to be more uniform when the values decreased to less than 2 μm (Figure 5g–l). In contrast to the 1.1 mm offset, the maximum thickness of the IMCs for a 1.2 mm offset existed at the bottom of the weld, rather than the middle. According to [10], welding–brazing joints have higher mechanical properties when the thickness of the IMCs is less than 5 μm. The mechanical properties of Ti/Al dissimilar joints increased due to the uniform distribution of the IMCs. In the welding–brazing process of Ti/Al dissimilar metals, the Ti alloy was almost infusible and only a very small amount of titanium could react with aluminum in the weld to form IMCs. Therefore, the most common IMC is TiAl_3_. However, when the titanium alloy melts excessively, the reaction of titanium and aluminum is intensified, forming TiAl_2_ and Ti_2_Al_5_ in the IMC layer. This leads to a decrease of the mechanical properties of the weld joint, due to increasing brittleness of the interface layer.

### 3.4. Hardness Values 

**Figure 6:**
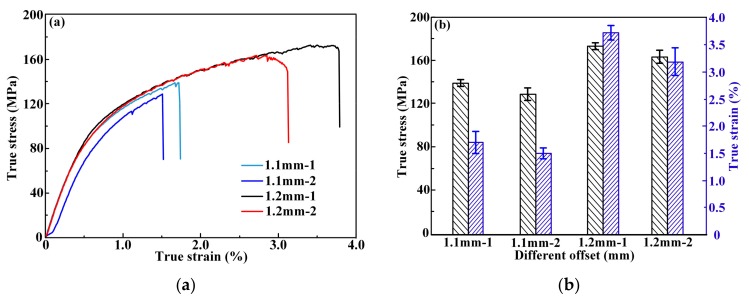
Tensile properties of Ti/Al joints: (**a**) true stress–strain curves; (**b**) tensile strength and fracture elongation.

The tensile properties of the Ti/Al welding–brazing joints are shown in Figure 6. The tensile properties of the welded joints were found to be sensitive to the laser offset. The thickness and distribution of the IMCs was quite different for different laser offsets. It is evident from Figure 6a that the joints had a lower tensile strength (139 and 128 MPa for oscillation frequencies of 28 and 30 Hz, respectively) for a laser offset of 1.1 mm, whereas the tensile strength significantly increased to 173 and 164 MPa for oscillation frequencies of 28 and 30 Hz, respectively (Figure 6b). As discussed earlier, with a 1.1 mm laser offset, the TC4 alloy underwent micro melting, which resulted in a large number of IMCs with uneven thickness during welding. IMCs with large size or uneven thickness significantly reduced the mechanical performance of the brazed joint, whereas in the fusion zone the porosity defect was the main cause of reduced mechanical properties. With a laser offset of 1.2 mm, the IMCs were distributed with uniform thickness (<5 μm) and had less porosity defects. These two factors resulted in a significant increase in tensile strength.

**Figure 7:**
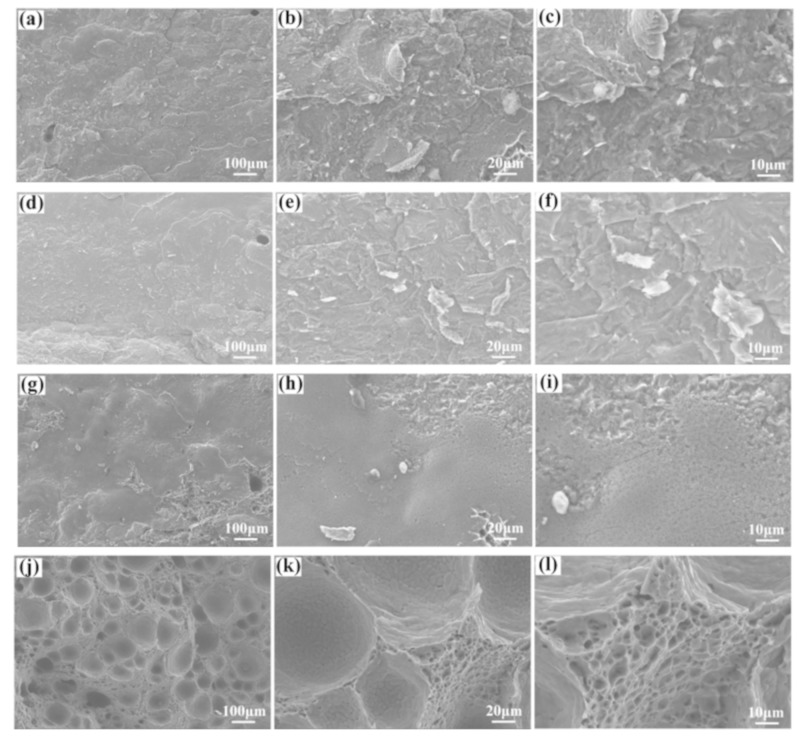
Tensile fractures of the welded joints: (**a**–**c**) 1#; (**d**–**f**) 2#; (**g**–**i**) 3#; (**j**–**l**) 4#.

The fracture morphologies of the Ti/Al dissimilar metal joints are shown in Figure 7. As mentioned above, a large number of IMCs formed at the surface of the TC4 alloy when the laser offset was 1.1 mm. The tensile specimens tended to fracture on the brazing side due to the formation of brittle IMCs. The tensile fracture surfaces were observed to be relatively flat with few existing cleavage steps (Figure 7a–f) for a laser offset of 1.1 mm, whereas some white phases were found to be randomly distributed on the fracture surfaces as observed in the magnified images (Figure 7c,f). When the laser offset was 1.2 mm, even though the size of the IMCs on the fracture surface decreased significantly, the tensile specimens were still fractured on the side of the brazed joints as seen in Figure 7g–i. The tensile properties of the Ti/Al joints increased with the decrease of porosity and the IMC size. At a frequency of 30 Hz and a laser offset of 1.2 mm, some porosity defects were formed in the fusion zone, which was a weak zone in the joint, and the tensile specimen tended to fracture in this zone. Figure 7j–l shows that there were some porosity defects on the fracture surface. The size of porosity was spherical and the inner wall of the porosity was smooth, which indicated that it was metallurgical porosity. Due the increase of porosity defects, the tensile properties of the welded joints were reduced.

**Figure 8:**
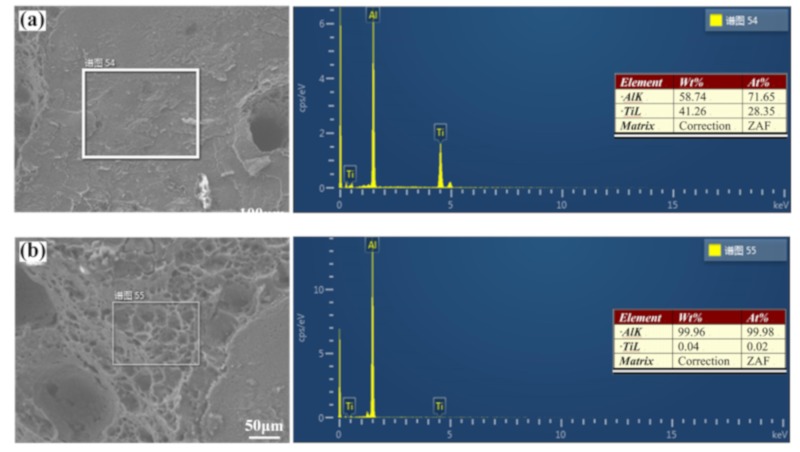
Fracture positions in Ti/Al joints produced by different offsets: (**a**) 1.1 mm—30 Hz; (**b**) 1.2 mm—30 Hz.

The EDS results of tensile fracture at different laser offsets are given in Figure 8. When the laser offset and oscillation frequency were 1.1 mm and 30 Hz, the element distribution of the fracture surface was 58.74 wt% Ti and 41.26 wt% Al, indicating fracture of the tensile specimen at the brazing zone. However, with a 1.2 mm laser offset and an oscillation frequency of 30 Hz, the element distribution was mainly Al. The tensile specimen fractured at the fusion zone and the experimental results showed good consistency.

## 4. Conclusions

Ti/Al butt joints were welded by the laser oscillation method, and their corresponding formation, microstructure, and tensile properties were studied in this manuscript. With a laser offset of 1.2 mm and oscillation frequencies of 28 and 30 Hz, the obtained Ti/Al dissimilar joints had excellent IMCs distribution and mechanical properties. Through analysis of the experimental results, the following conclusions can be drawn:

(1) With a laser offset of 1.1 mm and an oscillation frequency of 30 Hz, the Ti6Al4V alloy was micro melted, resulting in a large number of IMCs and porosity defects in the welds. The thicknesses of the IMCs in the upper, middle, and lower positions of the weld were 3.1, 4.9, and 1.7 μm, respectively. These changed to 5.6, 7.4, and 1.6 μm, respectively, when the oscillation frequency was decreased to 25 Hz. When the laser offset was increased to 1.2 mm, the thicknesses of the IMCs at different positions in the welds were less than 2 μm.
(2) With a 1.1 mm laser offset, the tensile strength of joints was 139 and 128 MPa for the oscillation frequencies of 30 and 25 Hz, respectively. When the laser offset changed from 1.1 to 1.2 mm, the tensile strength of the joints increased to 173 and 164 MPa with 30 and 25 Hz oscillation frequencies, respectively.
(3) With a 1.1 mm laser offset, the tensile specimens fractured along the Ti6Al4V surface due to the mass formation of brittle IMCs. When the laser offset increased to 1.2 mm, the tensile specimens tended to fracture at the FZ with an oscillation frequency of 30 Hz. The tensile strength of welded dissimilar joints can be significantly improved with the uniform distribution of IMCs.

## Figures and Tables

**Table 1:** Process parameters of laser welding of Ti/Al with beam oscillation.

Number	Frequency (Hz)	Offset (mm)	Laser Power (kW)	
			Al Side	Ti Side
1#	30	1.1	3.4, 3.2, 3.2, 3.0, 3.2, 3.2, 3.4	0.3, 0.2, 0.1, 0.2, 0.3
2#	25	1.1	3.2, 3.0, 3.0, 2.8, 3.0, 3.0, 3.2	1.5, 0.2, 0.1, 0.2, 0.3
3#	28	1.2	3.4, 3.8, 3.8, 3.8, 3.8, 3.8, 3.4, 2.5	1.5, 0.1, 0.1, 0.3
4#	30	1.2	3.5, 3.5, 3.5, 3.4, 3.4, 3.5, 3.5, 1.5	0.1, 0.1, 0.2, 1.5
